# Association between red cell distribution width-to-albumin ratio and all-cause mortality in critically ill cirrhotic patients with sepsis: a retrospective analysis of the MIMIC-IV database

**DOI:** 10.3389/fmed.2025.1610726

**Published:** 2025-06-25

**Authors:** Jinfeng Li, Shifeng Pang, Huiya Huang, Yangni Lu, Tingting Tang, Jianlin Wu, Maowei Chen

**Affiliations:** ^1^Department of Infectious Diseases, Wuming Hospital Affiliated to Guangxi Medical University, Nanning, Guangxi, China; ^2^Department of Cardiology, Minzu Hospital of Guangxi Zhuang Autonomous Region, Nanning, Guangxi, China; ^3^Department of General Medicine, Wuming Hospital Affiliated to Guangxi Medical University, Nanning, Guangxi, China

**Keywords:** cirrhosis, sepsis, all-cause mortality, red cell distribution width-to-albumin ratio (RAR), prognosis

## Abstract

**Background:**

Critically ill cirrhotic patients are at high risk of infections, which are associated with significantly increased mortality. The red cell distribution width-to-albumin ratio (RAR) is a validated predictor of mortality in critically ill patients. However, the prognostic value of RAR in critically ill cirrhotic patients with sepsis has not been fully established.

**Methods:**

This study retrospectively analyzed data from the Medical Information Mart for Intensive Care (MIMIC-IV) database. Patients were stratified into quartiles based on RAR values. The primary outcomes were 30-day and 365-day all-cause mortality. Kaplan–Meier survival analysis and multivariable Cox regression models were applied to assess the association between RAR and mortality. Restricted cubic spline (RCS) analysis confirmed a linear relationship and subgroup analyses explored potential interactions.

**Results:**

A total of 2,100 patients were included. Elevated RAR values were significantly associated with increased 30-day and 365-day all-cause mortality. Compared with the lowest quartile, patients in the highest RAR quartile had a 51% higher risk of 30-day mortality [hazard ratio (HR) = 1.51, 95% confidence interval (CI): 1.19–1.92) and a 51% higher risk of 365-day mortality (HR = 1.51, 95% CI: 1.25–1.81). RCS analysis confirmed a significant linear relationship between RAR and mortality risk. Subgroup analyses showed a stronger association between RAR and mortality in elderly patients.

**Conclusion:**

In critically ill cirrhotic patients with sepsis, elevated RAR values are independently associated with increased all-cause mortality risk. This study highlights the potential of RAR as a prognostic biomarker, particularly in elderly patients.

## 1 Introduction

Cirrhosis, the terminal stage of chronic progressive liver disease, contributes to approximately 1 million deaths globally each year (1). In cirrhosis, both internal factors (e.g., immune dysfunction) and external factors (e.g., alcohol consumption and invasive procedures) contribute to increased susceptibility to co-infections and disease progression. Approximately two-thirds of cirrhotic patients with peripheral organ failure develop sepsis (2–4). Infections quadruple the mortality risk in patients with cirrhosis, with 30% dying within 1 month and another 30% within 1 year (5). Despite advances in intensive care management for patients with cirrhosis, mortality rates remain high (6). The severity and prognosis of cirrhosis can be assessed using scoring systems such as the Child-Turcotte-Pugh score (7), CLIF-C ACLF score (8), and MELD score and its derivatives scoring system (9, 10). However, these scoring systems seem less effective when cirrhosis is complicated by infection. Although scores like the Sequential Organ Failure Assessment (SOFA) score (11), the Model for End-stage Liver Disease with the incorporation of serum sodium (MELD-Na) (12), and Age-Bilirubin-International Normalized Ratio (INR)-Creatinine (ABIC) score (13) can be used for prognosis assessment in cirrhotic patients with sepsis, their predictive value is limited due to the specificity of cirrhotic patients with sepsis, and their performance is still suboptimal.

Red blood cell distribution width (RDW), a simple, low-cost, and widely available parameter, reflects red blood cell volume heterogeneity (14). RDW has been shown to reflect systemic inflammation in critically ill patients and is a reliable predictor of sepsis risk (15). It has been reported that RDW can predict short-term mortality in critically ill patients with chronic obstructive pulmonary disease (COPD) and atrial fibrillation (AF) (16, 17). Serum albumin, an important protein synthesized by the liver, binds to inflammatory mediators and reflects inflammation severity (18, 19). Evidence suggests that low serum albumin levels are associated with higher mortality in patients with decompensated cirrhosis and sepsis (20, 21). Combining these parameters has led to the development of a novel inflammatory biomarker: the red cell distribution width-to-albumin ratio (RAR). Studies have shown that RAR can be used as an important prognostic indicator in critically ill patients with sepsis (22), after burn surgery (23), diabetic foot ulcers (24), acute respiratory distress syndrome (25), and rheumatic diseases (26). However, the association between RAR and outcomes in critically ill cirrhotic patients with sepsis remains incompletely understood. This study aims to investigate the relationship between RAR and all-cause mortality in these patients.

## 2 Materials and methods

### 2.1 Data source

A retrospective analysis was conducted using the Medical Information Mart for Intensive Care (MIMIC-IV) database (version 3.0), which contains a comprehensive and high-granularity dataset of well-defined patients admitted to the intensive care unit (ICU) of Beth Israel Deaconess Medical Center (BIDMC) between 2008 and 2022 (27). To obtain the qualification to use this database, the first author of this study, Jinfeng Li, completed the Collaborative Institutional Training Initiative (CITI) course and passed both the “Conflicts of Interest” and “Data or Specimens Only Research” exams (Record ID: 14347715). The database was approved by the institutional review boards of the Massachusetts Institute of Technology (Cambridge, MA, USA) and BIDMC (Boston, MA, USA) (27). The BIDMC institutional review board waived informed consent due to the use of anonymized and publicly available data, and the sharing of study resources was approved.

### 2.2 Study design and population

**FIGURE 1:**
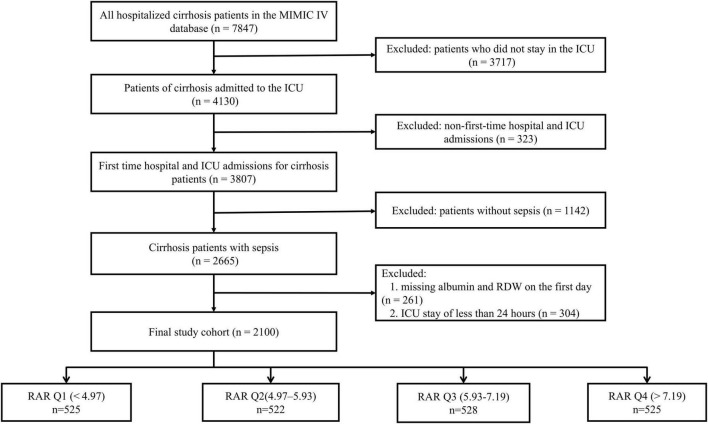
The flow chart of participants.

This study included adult patients (age > 18 years) diagnosed with sepsis and severe liver cirrhosis. Liver cirrhosis was diagnosed based on International Classification of Diseases, Ninth Revision (ICD-9) codes (5712, 5715, and 5716) and Tenth Revision (ICD-10) codes (K7469, K7031, K7030, K7460, K743, K745, K744, K717, and K741). Sepsis was defined according to the Sepsis-3 criteria, i.e., infection combined with a SOFA score ≥2 (28). The method for screening patients meeting the Sepsis-3 criteria from the MIMIC database was consistent with previous studies (29). Exclusion criteria were as follows: (1) patients with an ICU stay <1 day; (2) patients with multiple ICU admissions for sepsis and severe liver cirrhosis, with only the first admission included; and (3) patients with missing values for RDW or serum albumin. A total of 2,100 patients were included and divided into four groups based on RAR index quartiles (Figure 1).

### 2.3 Demographic and laboratory variables

We used Structured Query Language (SQL) via Navicat Premium (version 15.0.12) to extract data from the MIMIC-IV database. Extracted data included demographics (age, gender, race, height, and weight), vital signs (heart rate, respiratory rate, and mean arterial pressure), laboratory values (hemoglobin, white blood cell count, serum albumin, etc.), comorbidities [heart failure and acute kidney injury (AKI)], clinical severity scores [Acute Physiology Score III (APS III), SOFA score, etc.], and information on vasopressor use and invasive mechanical ventilation (IMV). For variables measured multiple times, the first recorded value was used. Missing data were handled using multiple imputations, and variables with missing values exceeding 20% were excluded.

### 2.4 RAR assessment and outcomes

The RAR was calculated as RDW (%) divided by serum albumin (g/dl). Outcomes included all-cause mortality at 30, 90, 180, and 365 days. Primary outcomes were 30-day and 365-day all-cause mortality, while secondary outcomes were 90-day and 180-day all-cause mortality.

### 2.5 Statistical analysis

Continuous variables were tested for normality. Normally distributed data were analyzed using Student’s *t*-test and one-way ANOVA and presented as mean ± SD. Non-normally distributed data were analyzed using the Wilcoxon rank-sum test and presented as median (IQR). Categorical variables, expressed as absolute numbers and percentages, were analyzed using the Chi-square test or Fisher’s exact test. Patients were stratified into quartiles 1–4 according to the LAR quartile. Kaplan–Meier (K-M) curves were used to evaluate the incidence of primary and secondary outcomes. The association between the RAR index and primary outcomes was assessed using Cox proportional hazards models, with adjustments for multiple covariates. To avoid overfitting due to multicollinearity, variance inflation factors (VIFs) were calculated, and variables with VIF ≥ 10 were excluded. Clinical and prognostic-related variables were included in the multivariable models: model 1 included only the RAR index; model 2 adjusted for age, gender, race, and BMI; and model 3 further adjusted for heart failure, AKI, white blood cell count, red blood cell count, hematocrit, chloride, glucose, anion gap, lactate, partial thromboplastin time (PTT), total bilirubin, alanine aminotransferase (ALT), SOFA score, APS III score, Charlson score, ICU length of stay, liver transplantation, IMV, and vasopressor use. The RAR index was entered into the models both as a continuous variable and as a categorical variable (with the lowest quartile as the reference group). The *P* for trend was calculated using linear regression analysis by converting RAR quartiles into their respective median values. The RAR index was analyzed as a continuous variable using restricted cubic splines (RCS) to clarify the dose-effect correlations with the risk of major and secondary outcome events. Subgroup analyses were performed based on gender, age (<60 years or ≥60 years), race, heart failure, and AKI.

All statistical analyses were performed using SPSS software (version 23.0, IBM Corporation, USA) and R software (version 4.2.2, R Foundation).^1^

## 3 Results

### 3.1 Baseline characteristics of study subjects

A total of 2,100 critically ill cirrhotic patients with sepsis were included in the analysis. The mean age of participants was 59.00 (52.00, 67.00) years, and 64.29% were male. Baseline characteristics of study participants, stratified by RAR quartiles at admission (Q1: <4.97, Q2: 4.97–5.93, Q3: 5.93–7.19, and Q4: >7.19), are presented in Table 1. Patients in the high RAR group exhibited higher heart rate, WBC, RDW, PTT, AST, lactate, total bilirubin, SOFA, APS III, SAPS II, OASIS, and SIRS scores, but lower age, red blood cell count, hemoglobin, albumin, sodium, chloride, glucose, and anion gap. The 30-day, 90-day, 180-day, and 365-day all-cause mortality rates were 30.86%, 40.62%, 44.95%, and 50.38%, respectively, with higher mortality observed in the high RAR group. The mortality rate increased in a dose-dependent manner with higher RAR quartiles at all time points. The 30-day mortality rate rose from 23.81% in Q1 to 39.62% in Q4, corresponding to an Absolute Risk Increase (ARI) of +4.73%, +2.90%, and +8.18% per quartile. Similarly, the 365-day mortality rate increased from 43.43% in Q1 to 60.19% in Q4, with an ARI per quartile of +2.55%, +5.91%, and +8.30%. The detailed results are presented in Table 1.

**TABLE 1:** Baseline characteristics of patients grouped according to RAR index quartiles.

Variables	Total (*N* = 2,100)	Q1 < 4.97 (*N* = 525)	Q2 4.97–5.93 (*N* = 522)	Q3 5.93–7.19 (*N* = 528)	Q4 > 7.19 (*N* = 525)	*P*
General characteristics						
Gender (male), *n* (%)	1,350 (64.29)	353 (67.24)	339 (64.94)	340 (64.39)	318 (60.57)	0.154
Race (white), *n* (%)	1,614 (76.86)	411 (78.29)	400 (76.63)	406 (76.89)	397 (75.62)	0.293
Age (years)	59.00 (52.00, 67.00)	60.00 (53.00, 68.00)	59.00 (53.00, 67.00)	59.00 (51.00, 66.00)	58.00 (50.00, 66.00)	0.005
BMI (kg/m^2^)	29.79 (26.96, 30.58)	29.79 (27.33, 30.61)	29.79 (27.05, 30.21)	29.79 (26.82, 30.62)	29.79 (26.70, 30.85)	0.914
Vital signs						
Heart rate (beats/min)	93.00 (80.00, 107.00)	90.00 (76.00, 103.00)	92.00 (78.00, 107.00)	93.00 (81.00, 107.00)	95.00 (82.00, 109.00)	<0.001
Respiratory rate (breaths/min)	19.00 (16.00, 23.00)	19.00 (16.00, 23.00)	19.00 (16.00, 22.00)	19.00 (16.00, 23.00)	20.00 (16.00, 24.00)	0.138
MBP (mmHg)	78.00 (67.00, 90.00)	78.00 (67.00, 93.00)	78.00 (67.00, 90.00)	77.00 (68.00, 89.25)	77.00 (66.00, 87.00)	0.106
Laboratory parameters						
WBC (10^9^/L)	10.30 (6.50, 15.80)	9.80 (6.40, 13.90)	10.05 (6.60, 15.38)	10.10 (6.10, 16.20)	11.60 (7.20, 18.30)	<0.001
RBC (10^12^/L)	2.96 (2.52, 3.46)	3.10 (2.59, 3.66)	3.06 (2.62, 3.55)	2.92 (2.49, 3.40)	2.78 (2.43, 3.24)	<0.001
Hemoglobin (g/dl)	9.30 (8.00, 10.70)	9.80 (8.40, 11.40)	9.60 (8.30, 10.90)	9.10 (7.88, 10.60)	8.80 (7.60, 10.10)	<0.001
RDW (%)	17.00 (15.28, 19.10)	15.10 (14.20, 16.50)	16.30 (15.10, 17.98)	17.50 (16.20, 19.22)	19.20 (17.20, 21.60)	<0.001
Hematocrit (%)	27.90 (24.30, 32.40)	29.40 (25.40, 34.20)	28.70 (25.13, 33.08)	27.30 (23.70, 31.50)	26.90 (23.20, 30.70)	<0.001
Albumin (g/dl)	2.90 (2.50, 3.30)	3.50 (3.20, 3.90)	3.00 (2.80, 3.30)	2.70 (2.50, 3.00)	2.30 (2.00, 2.50)	<0.001
Sodium (mmol/L)	137.00 (132.00, 140.00)	137.00 (132.00, 140.00)	138.00 (134.00, 141.00)	137.00 (132.00, 140.00)	136.00 (132.00, 139.00)	<0.001
Potassium (mmol/L)	4.20 (3.70, 4.70)	4.20 (3.80, 4.70)	4.10 (3.70, 4.70)	4.20 (3.70, 4.80)	4.10 (3.60, 4.80)	0.075
Chloride (mmol/L)	103.00 (97.00, 107.00)	102.00 (97.00, 106.00)	103.00 (98.25, 107.00)	103.00 (97.00, 108.00)	102.00 (98.00, 107.00)	0.002
Glucose (mg/dl)	127.00 (102.00, 167.00)	129.00 (105.00, 173.00)	130.00 (104.25, 176.75)	124.00 (102.00, 164.00)	122.00 (97.00, 158.00)	0.003
Anion gap (mmol/L)	15.00 (12.00, 19.00)	16.00 (13.00, 20.00)	15.00 (12.00, 19.00)	15.00 (12.00, 18.00)	14.00 (12.00, 18.00)	<0.001
Ph	7.35 (7.31, 7.41)	7.35 (7.31, 7.41)	7.36 (7.31, 7.42)	7.36 (7.31, 7.41)	7.35 (7.30, 7.42)	0.215
Lactate (mmol/L)	2.60 (1.70, 3.60)	2.50 (1.60, 3.38)	2.50 (1.70, 3.48)	2.70 (1.70, 3.70)	2.80 (1.80, 4.10)	0.009
PTT (s)	37.85 (32.00, 47.20)	37.20 (31.20, 49.40)	36.50 (31.60, 45.08)	37.20 (32.18, 45.30)	41.00 (33.80, 50.40)	<0.001
Total bilirubin (mg/dl)	3.30 (1.50, 8.00)	2.30 (1.10, 5.20)	2.90 (1.40, 6.70)	3.90 (1.80, 8.53)	4.90 (2.00, 10.50)	<0.001
ALT (U/L)	36.00 (21.00, 88.00)	33.00 (19.00, 80.00)	37.00 (21.00, 108.50)	34.50 (21.00, 80.00)	38.00 (22.00, 88.00)	0.113
AST (U/L)	76.00 (42.00, 181.00)	60.00 (37.00, 151.00)	74.00 (42.00, 199.25)	79.00 (44.00, 171.00)	91.00 (46.00, 212.00)	<0.001
Comorbidities						
Heart failure	336 (16.00)	92 (17.52)	87 (16.67)	74 (14.02)	83 (15.81)	0.450
AKI	1,347 (64.14)	336 (64.00)	332 (63.60)	335 (63.45)	344 (65.52)	0.892
Disease severity scores						
SOFA (scores)	9.00 (6.00, 12.00)	9.00 (6.00, 11.00)	8.50 (6.00, 11.00)	9.00 (7.00, 12.00)	9.00 (7.00, 12.00)	<0.001
APS III (scores)	60.00 (45.00, 77.00)	54.00 (40.00, 74.00)	55.00 (42.00, 72.75)	60.00 (46.00, 77.00)	66.00 (53.00, 84.00)	<0.001
SAPS II (scores)	41.00 (32.00, 51.00)	40.00 (31.00, 50.00)	41.00 (32.00, 49.00)	42.00 (32.00, 51.25)	44.00 (34.00, 55.00)	<0.001
OASIS (scores)	34.00 (28.00, 40.00)	33.00 (27.00, 39.00)	33.00 (28.00, 39.00)	34.00 (28.00, 41.00)	36.00 (30.00, 42.00)	<0.001
SIRS (scores)	3.00 (2.00, 3.00)	3.00 (2.00, 3.00)	3.00 (2.00, 3.00)	3.00 (2.00, 3.00)	3.00 (2.00, 3.00)	<0.001
CCI (scores)	6.00 (4.00, 8.00)	6.00 (4.00, 7.00)	6.00 (4.00, 8.00)	5.00 (4.00, 7.00)	6.00 (4.00, 8.00)	0.870
Medication or procedures on the first day of ICU admission						
Vasoactive agent, *n* (%)	994 (47.33)	233 (44.38)	242 (46.36)	262 (49.62)	257 (48.95)	0.296
IMV, *n* (%)	1,118 (53.24)	273 (52.00)	275 (52.68)	293 (55.49)	277 (52.76)	0.679
Length of stay (LOS)						
LOS in hospital	12.86 (6.97, 23.66)	12.31 (6.68, 23.08)	12.71 (7.45, 23.30)	12.71 (6.52, 22.91)	13.54 (7.68, 24.90)	0.404
LOS in ICU	3.83 (2.08, 7.80)	3.86 (1.92, 7.71)	3.79 (2.16, 7.82)	3.76 (2.14, 7.76)	3.87 (2.11, 7.89)	0.720
Liver transplantation (%)	160 (7.62)	37 (7.05)	38 (7.28)	42 (7.95)	43 (8.19)	0.883
Outcomes						
30-day mortality, *n* (%)	648 (30.86)	125 (23.81)	149 (28.54)	166 (31.44)	208 (39.62)	<0.001
90-day mortality, *n* (%)	853 (40.62)	180 (34.29)	195 (37.36)	212 (40.15)	266 (50.67)	<0.001
180-day mortality, *n* (%)	944 (44.95)	206 (39.24)	213 (40.80)	232 (43.94)	293 (55.81)	<0.001
365-day mortality, *n* (%)	1,058 (50.38)	228 (43.43)	240 (45.98)	274 (51.89)	316 (60.19)	<0.001

BMI, body mass index; MBP, mean blood pressure; WBC, white blood cell; RBC, red blood cell; RDW, red blood cell distribution width; PTT, partial thromboplastin time; ALT, alanine aminotransferase; AST, aspartate transaminase; AKI, acute kidney injury; SOFA, Sequential Organ Failure Assessment score; APS III, acute physiology score III; SAPS II, simplified acute physiology score II; OASIS, oxford acute severity of illness score; SIRS, systemic inflammatory response syndrome score; CCI, Charlson Comorbidity Index; IMV, invasive mechanical ventilation; ICU, intensive care unit.

### 3.2 Association between RAR and mortality

**FIGURE 2:**
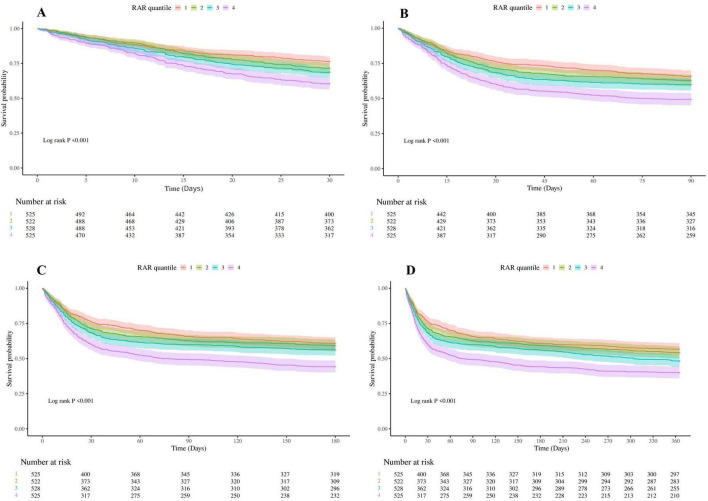
Kaplan–Meier analysis of critically ill cirrhotic patients with sepsis according to RAR quartiles (**A**, 30-day mortality; **B**, 90-day mortality; **C**, 180-day mortality; **D**, 365-day mortality). RAR, red blood cell distribution width-to-albumin ratio. RAR quartiles: Q1: <4.97, Q2: 4.97–5.93, Q3: 5.93–7.19, and Q4: >7.19.

Kaplan–Meier survival analysis (Figure 2) revealed significant differences in survival probabilities across RAR quartiles (all log-rank *P* < 0.001). Survival rates at 30, 90, 180, and 365 days decreased significantly with increasing RAR quartiles.

Three Cox regression models were developed to evaluate the independent effect of the RAR index on mortality (Table 2). When treated as a continuous variable, RAR was significantly associated with increased risk of mortality at 30, 90, 180, and 365 days (*P* < 0.001), with hazard ratios (HRs) indicating a 7%, 8%, 8%, and 8% increase in mortality risk, respectively. Even after adjusting for confounders in model 3, RAR remained significantly associated with mortality (*P* < 0.001), with a 9% increase in mortality risk at all time points. A consistent trend was observed when RAR was categorized into quartiles, with higher RAR quartiles corresponding to higher all-cause mortality risks (*P* for trend < 0.001). After adjusting for model 2 and model 3, the consistent trend remained (*P* for trend < 0.001). Specifically, even after model 3 adjustment, mortality risk across all time points increased stepwise with higher RAR quartiles. Each quartile increase was associated with a 10%–26% rise in mortality risk. Notably, the Q4 group exhibited a 49% to 51% higher risk than the Q1 group. Variance inflation factors (VIFs) for covariates were all below 10, indicating minimal multicollinearity and robustness of the multivariable models.

**TABLE 2:** Relationship between RAR and mortality in critically ill cirrhotic patients with sepsis.

Variables	Model 1		Model 2		Model 3	
	HR (95% CI)	*P*	HR (95% CI)	*P*	HR (95% CI)	*P*
30-day mortality						
RAR	1.07 (1.05 ∼ 1.10)	<0.001	1.07 (1.04 ∼ 1.09)	<0.001	1.09 (1.05 ∼ 1.13)	<0.001
RAR quantile						
1	1.00 (reference)		1.00 (reference)		1.00 (reference)	
2	1.22 (0.96 ∼ 1.54)	0.106	1.24 (0.98 ∼ 1.58)	0.072	1.21 (0.95 ∼ 1.55)	0.114
3	1.38 (1.09 ∼ 1.74)	0.006	1.45 (1.15 ∼ 1.83)	0.002	1.34 (1.05 ∼ 1.70)	0.017
4	1.85 (1.48 ∼ 2.31)	<0.001	1.99 (1.59 ∼ 2.49)	<0.001	1.51 (1.19 ∼ 1.92)	<0.001
*P* for trend		<0.001		<0.001		<0.001
90-day mortality						
RAR	1.08 (1.05 ∼ 1.10)	<0.001	1.07 (1.05 ∼ 1.09)	<0.001	1.09 (1.06 ∼ 1.13)	<0.001
RAR quantile						
1	1.00 (reference)		1.00 (reference)		1.00 (reference)	
2	1.12 (0.91 ∼ 1.37)	0.271	1.15 (0.94 ∼ 1.41)	0.183	1.18 (0.96 ∼ 1.45)	0.116
3	1.25 (1.02 ∼ 1.52)	0.028	1.31 (1.07 ∼ 1.60)	0.008	1.28 (1.04 ∼ 1.57)	0.019
4	1.71 (1.41 ∼ 2.07)	<0.001	1.86 (1.54 ∼ 2.25)	<0.001	1.49 (1.21 ∼ 1.83)	<0.001
*P* for trend		<0.001		<0.001		<0.001
180-day mortality						
RAR	1.08 (1.05 ∼ 1.10)	<0.001	1.07 (1.05 ∼ 1.09)	<0.001	1.09 (1.06 ∼ 1.13)	<0.001
RAR quantile						
1	1.00 (reference)		1.00 (reference)		1.00 (reference)	
2	1.07 (0.88 ∼ 1.30)	0.487	1.10 (0.91 ∼ 1.33)	0.335	1.13 (0.93 ∼ 1.38)	0.207
3	1.20 (0.99 ∼ 1.44)	0.060	1.26 (1.04 ∼ 1.52)	0.018	1.23 (1.01 ∼ 1.49)	0.037
4	1.67 (1.40 ∼ 2.00)	<0.001	1.83 (1.53 ∼ 2.19)	<0.001	1.49 (1.23 ∼ 1.80)	<0.001
*P* for trend		<0.001		<0.001		<0.001
365-day mortality						
RAR	1.08 (1.06 ∼ 1.10)	<0.001	1.07 (1.05 ∼ 1.09)	<0.001	1.09 (1.06 ∼ 1.13)	<0.001
RAR quantile						
1	1.00 (reference)		1.00 (reference)		1.00 (reference)	
2	1.09 (0.91 ∼ 1.31)	0.350	1.12 (0.93 ∼ 1.34)	0.217	1.17 (0.97 ∼ 1.40)	0.101
3	1.29 (1.08 ∼ 1.53)	0.005	1.35 (1.13 ∼ 1.61)	<0.001	1.32 (1.10 ∼ 1.58)	0.003
4	1.66 (1.40 ∼ 1.97)	<0.001	1.83 (1.54 ∼ 2.17)	<0.001	1.51 (1.25 ∼ 1.81)	<0.001
*P* for trend		<0.001		<0.001		<0.001

HR, hazard ratio; CI, confidence interval; RAR, red cell distribution width to albumin ratio; BMI, body mass index; WBC, white blood cell; RBC, red blood cell; PTT, partial thromboplastin time; ALT, alanine aminotransferase; AKI, acute kidney injury; SOFA, Sequential Organ Failure Assessment score; APS III, acute physiology score III; CCI, Charlson Comorbidity Index; IMV, invasive mechanical ventilation; LOS, length of stay; ICU, intensive care unit. Model 1: crude. Model 2: adjust: gender, race, age, and BMI. Model 3: adjust: gender, race, age, BMI, heart failure, AKI, liver transplantation, IMV, vasoactive agent, WBC, RBC, hematocrit, chloride, glucose, anion gap, lactate, PTT, TBIL, ALT, SOFA, APS III, CCI, and LOS in ICU. RAR quartiles: Q1 < 4.97, 4.97 ≥ Q2 > 5.93, 5.93 ≥ Q3 ≤ 7.19, and Q4 > 7.19.

### 3.3 Linear associations

**FIGURE 3:**
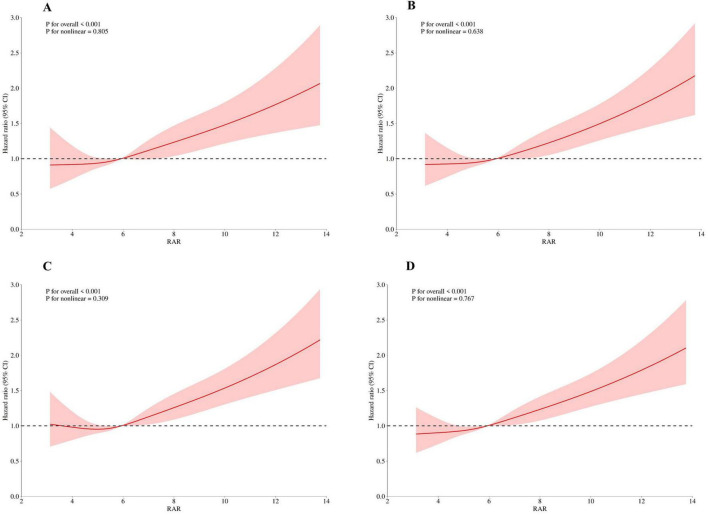
Restricted cubic spline analysis of mortality risk with RAR in critically ill cirrhotic patients with sepsis (**A**, 30-day mortality; **B**, 90-day mortality; **C**, 180-day mortality; **D**, 365-day mortality). RAR, red blood cell distribution width-to-albumin ratio.

Adjusted RCS models demonstrated a significant linear relationship between RAR and all-cause mortality at 30, 90, 180, and 365 days (Figures 3A–D). RCS curves indicated a significant linear correlation between RAR and 30-day mortality risk (*P* for non-linear = 0.805), with increasing RAR values associated with higher mortality risks. Similar linear associations were observed for 90-day, 180-day, and 365-day mortality (*P* for non-linear 0.638, 0.309, and 0.767, respectively).

### 3.4 Subgroup analyses of the relationship between RAR and mortality

**FIGURE 4:**
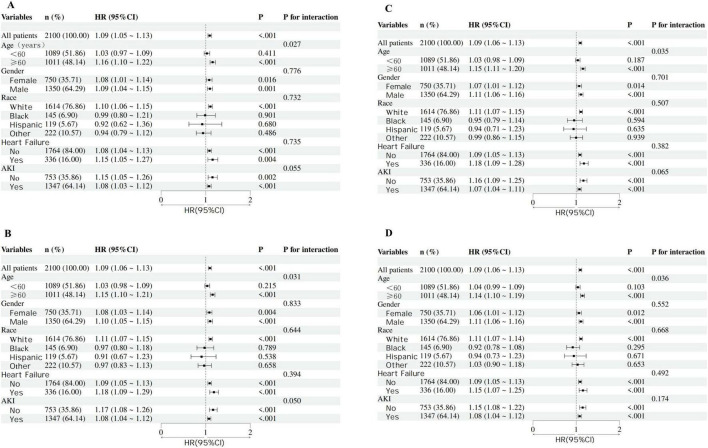
Forest plots of subgroup analysis of the association between RAR and mortality risk of critically ill cirrhotic patients with sepsis (**A**, 30-day mortality; **B**, 90-day mortality; **C**, 180-day mortality; **D**, 365-day mortality).

Adjusted forest plots (Figure 4) illustrated the association between RAR and mortality across different clinical characteristics. Specifically, a positive correlation between RAR and all-cause mortality was observed across various age groups, genders, races, AKI, and heart failure subgroups. Interaction tests indicated significant differences in the relationship between RAR and mortality across age subgroups. Patients aged ≥ 60 years exhibited a more pronounced positive correlation between RAR and mortality at 30, 90, 180, and 365 days compared to those aged < 60 years. No significant interactions were found in other subgroups.

## 4 Discussion

This study investigated the association between the red blood cell distribution width-to-albumin ratio (RAR) and mortality in critically ill cirrhotic patients with sepsis. Our analysis of data from the MIMIC-IV database revealed a strong association between elevated RAR and increased mortality risk. Kaplan–Meier survival analysis and multivariable Cox regression models consistently demonstrated that higher RAR quartiles were significantly associated with increased 30-day, 90-day, 180-day, and 365-day mortality. RCS analysis further confirmed a significant linear relationship between elevated RAR values and increased mortality risk. Subgroup analyses and interaction effects indicated a stronger association between RAR and mortality in older patients.

Red cell distribution width-to-albumin ratio, as a simple biochemical marker, has demonstrated superior predictive capabilities compared to the use of RDW and serum albumin alone (30, 31). Seo et al. (23) reported that RAR was associated with 90-day mortality in patients after burn surgery. Additionally, a study on patients with chronic kidney disease (CKD) found that higher RAR was significantly associated with CKD progression, all-cause mortality, and cardiovascular events (32). Furthermore, RAR has been identified as a robust prognostic indicator in patients with sepsis, septic patients with AF, and sepsis-related non-thyroidal illness syndrome (22, 33, 34). However, the relationship between RAR and outcomes in critically ill cirrhotic patients with sepsis has not been well-established. Our study, which included 2,100 critically ill cirrhotic patients with sepsis, demonstrated a significant association between RAR and both short-term and long-term mortality, with higher RAR values corresponding to worse clinical outcomes. These findings support the potential of RAR as a prognostic marker in this patient population.

The relationship between RAR and mortality in critically ill cirrhotic patients with sepsis involves complex mechanisms. During critically ill cirrhotic patients with sepsis, inflammatory cells generate reactive oxygen species (ROS), including hydrogen peroxide, superoxide anions, and hydroxyl radicals, which directly damage red blood cell membranes. Inflammatory cells also produce cytokines such as tumor necrosis factor α (TNFα) and interleukin 1β (IL-1β), which activate pathways leading to red blood cell apoptosis. These mechanisms result in the destruction of mature red blood cells and indirectly cause an elevated red cell distribution width (RDW) (35–37). Inflammatory activity can suppress iron metabolism and erythropoietin production (38), inhibiting red blood cell maturation and causing the release of immature red blood cells into the bloodstream, thereby increasing RDW levels. The decrease in serum albumin may be attributed to hepatic synthetic dysfunction (39) and capillary leakage caused by sepsis-related endothelial damage (22), both of which can significantly reduce serum albumin levels. RAR, which combines RDW and serum albumin, comprehensively reflects two pathological states: hematopoietic dysfunction and hypoalbuminemia (40–42). Our study showed a significant linear correlation between increased RAR values and higher mortality risk in critically ill cirrhotic patients with sepsis.

Subgroup analyses revealed a significant interaction between age and RAR as predictors of mortality. Elderly patients with elevated RAR exhibited a more pronounced increase in mortality risk. This interaction may stem from the higher comorbidity burden in elderly patients, combined with elevated RAR, leading to increased mortality risk. These findings underscore the importance of considering age as a modifier when evaluating the prognostic value of RAR. We interpret our subgroup analysis results with caution, as they may be influenced by heterogeneity across different populations. These results warrant further validation through additional studies.

Our study highlights the potential of RAR as a prognostic marker in critically ill cirrhotic patients with sepsis. Compared to existing prognostic scores such as the Child-Pugh score and the MELD score, RAR offers several advantages. It is a direct biochemical indicator of both hematological and hepatic function, with straightforward and low-cost measurement. Moreover, RAR avoids the subjectivity of certain markers (e.g., ascites and encephalopathy) in the Child-Pugh score and the laboratory heterogeneity of creatinine and INR measurements in the MELD score, as well as gender bias (43). By integrating inflammatory and nutritional markers, RAR provides a more comprehensive assessment of a patient’s physiological state (26, 44). In critically ill cirrhotic patients with sepsis, elevated RAR is linked to higher mortality risk. The stepwise rise in mortality risk with increasing RAR quartiles underscores its potential as a stratification tool for the rapid identification of high-risk patients. Additionally, RAR is easy to obtain and low-cost, making it a highly effective stratification tool for resource-limited settings. The use of data from the MIMIC-IV database ensures a large and diverse patient cohort, supporting robust statistical analysis. Our results indicate that RAR can serve as a valuable supplement to existing prognostic tools, particularly in critically ill patients where accurate prognosis is crucial for clinical decision-making.

While our study provides valuable insights into predicting mortality in critically ill cirrhotic patients with sepsis, it has limitations. First, our analysis is retrospective, limiting our ability to establish causality and potentially introducing biases inherent to observational studies. Second, our study cohort was derived from a single center. This limits the generalizability of our findings to diverse populations and healthcare settings. Although methodological safeguards were implemented, we cannot rule out the possibility of overfitting or chance findings. External validation was not performed due to study limitations, so our results should be interpreted with caution. Third, despite adjusting for numerous confounders, unmeasured variables such as laboratory test results, genetic factors, lifestyle, and specific treatments may still influence the relationship between RAR and mortality. Fourth, due to missing data common in retrospective studies, we were unable to include all metrics for the MELD score or the ACLF score in our analyses, and these will be considered in future prospective studies. Despite these limitations, our study contributes to the growing body of evidence highlighting the importance of RAR in managing critically ill cirrhotic patients with sepsis.

## 5 Conclusion

In critically ill cirrhosis patients with sepsis admitted to the ICU, elevated RAR is associated with increased short-term and long-term mortality. Thus, RAR measurement may aid in the prognostic management of these patients. Further prospective studies are needed to confirm our findings.

## Data Availability

Publicly available datasets were analyzed in this study. This data can be found here: https://physionet.org/content/mimiciv/3.0/.

## Footnotes

1
http://www.R-project.org
